# Analysis of changes in respiratory tract infections in a tertiary grade A hospital in Zhangjiakou area from 2018 to 2024

**DOI:** 10.1097/MD.0000000000043435

**Published:** 2025-07-18

**Authors:** Lei Zhang, Junshuai Ma, Shaobo Qu, Zhangxin Cui, Mingfu Cao

**Affiliations:** a Department of Clinical Laboratory, The First Affiliated Hospital of Hebei North University, Zhang Jiakou, Hebei Province, China; b Department of Traditional Chinese Medicine, The First Affiliated Hospital of Hebei North University, Zhang Jiakou, Hebei Province, China; c Department of General Surgery, The First Affiliated Hospital of Hebei North University, Zhang Jiakou, Hebei Province, China.

**Keywords:** epidemiology, influenza B virus, legionella pneumophila, mycoplasma pneumoniae, respiratory tract infection

## Abstract

We aimed to analyze the epidemiological characteristics and pathogen trends of common respiratory infections in a tertiary hospital in Zhangjiakou from 2018 to 2024. We retrospectively studied patients who underwent screening for 11 common respiratory pathogen antibodies at the First Affiliated Hospital of Hebei North University between January 1, 2018, and December 31, 2024. Serum-specific Immunoglobulin M antibodies were detected using indirect immunofluorescence assays, and annual data from 2018 to 2024 were compared. Among 35,665 patients, 10,531 (29.53%) were positive for at least 2 pathogen. The lowest positivity rate was observed in 2020 (23.47%, 841/3584), while the highest was observed in 2023 (38.58%, 3165/8204). Mycoplasma pneumoniae exhibited the highest positivity rate (11.99%, 4278/35,665), followed by influenza B virus (10.83%, 3861/35,665). Influenza A virus, respiratory syncytial virus, parainfluenza virus, Chlamydia pneumoniae, Legionella pneumophila, Coxsackie A virus, Coxsackie B virus, Echovirus, and adenovirus had relatively low positivity rates, ranging from 0.88% to 4.97%. Positive cases were stratified as follows: 0 to <3 years (13.32%, 1403/10,531), 3 to <6 years (17.52%, 1845/10,531), 6 to <12 years (18.35%, 1932/10,531), 12 to <18 years (6.37%, 671/10,531), 18 to <60 years (15.86%, 1670/10,531), and ≥60 years (28.58%, 3010/10,531). The proportion of patients <12 years significantly decreased by 2024 compared to 2018 (*P* < .001), while the proportion of patients significantly increased in the ≥60 group (*P* < .001). Single-pathogen infection was the predominant type (68.73%, 7238/10,531), followed by dual-pathogen infections (23.26%, 2450/10,531). The proportion of single-pathogen cases decreased from 75.59% in 2018 to 60.32% in 2023, rebounding to 74.36% in 2024. On the contrary, the number of dual-pathogen cases increased from 18.46% in 2018 to 27.65% in 2023, and then decreased to 21.67% in 2024 (*P* < .001). From 2018 to 2024, the epidemiological features of common respiratory pathogens exhibited significant temporal variations, with reduced positivity rates during the Coronavirus Disease 2019 pandemic and a sharp resurgence after the pandemic. Routine pathogen screening provides critical data for regional respiratory infection prevention and control.

## 1. Introduction

Respiratory infections are among the most prevalent diseases globally, caused by diverse pathogens, including bacteria, fungi, viruses, and mycoplasma. These infections are primarily transmitted via airborne droplets, and children, the elderly population, and immunocompromised individuals are more susceptible to these pathogens. Currently, upper respiratory infections rank first in terms of global incidence (12.8 billion cases annually), while lower respiratory infections are the seventh leading cause of mortality worldwide (2.18 million deaths).^[1]^ Notably, the pathogen spectrum dynamically shifts across regions, environmental conditions, and temporal periods.^[2]^ Understanding these epidemiological trends is critical for effective prevention and control of respiratory infections.

This study analyzed epidemiological characteristics and pathogen positivity rates among patients with respiratory infection at our institution from 2018 to 2024, aiming to provide an evidence-based guide for clinical management and public health interventions.

## 2. Materials and methods

### 2.1. Study population

We retrospectively analyzed clinical data from patients who were screened for 11 common respiratory pathogen antibodies at the First Affiliated Hospital of Hebei North University between January 1, 2018 and December 31, 2024.

The inclusion criteria were as follows:

① Presence of respiratory symptoms (e.g., fever, cough, or sputum production);
② Completion of the 11-pathogen antibody panel.

The exclusion criteria were as follows:

① Incomplete clinical records;
② Duplicate test results from follow-up visits.

#### 2.1.1. Age stratification

Based on World Health Organization standards and the eighth edition of Pediatrics,^[3]^ patients were categorized into the following groups: 0 to <3 years (infancy/toddler group), 3 to <6 years (preschool group), 6 to <12 years (school-age group), 12 to <18 years (adolescent group), 18 to <60 years (adult group), ≥60 years (elderly group).

This study was approved by the Ethics Committee of the First Affiliated Hospital of Hebei North University, and informed consent was waived (Approval No. K2024112).

### 2.2. Laboratory methods

Using the respiratory pathogen spectrum antibody (Immunoglobulin M [IgM]) detection kit (EUROIMMUN Medizinische Labordiagnostika AG) to screen for 11 types of respiratory pathogens, including influenza A virus, influenza B virus, and respiratory syncytial virus, parainfluenza virus, mycoplasma pneumoniae, chlamydia pneumoniae, legionella pneumophila, coxsackie A virus, coxsackie B virus, echovirus, and adenovirus. The patient’s serum samples were diluted at a ratio of 1:10 for legionella pneumophila and at a ratio of 1:100 for mycoplasma pneumoniae. The samples were then incubated with biological slides coated with a detection substrate (antigen). When the sample was positive, the specific IgM antibody bound to the corresponding antigen. After adding Fluorescein isothiocyanate-labeled fluorescent antiantibody, the bound antibody will react with the fluorescent antiantibody. A specific fluorescence model was observed under the fluorescence microscope. The criterion for adjudicating the results was as follows: At the recommended dilution, if any one of the 11 common pathogen antibody tests showed specific fluorescence, it was considered positive. In the absence of specific fluorescence, it was regarded as negative.

### 2.3. Statistical analysis

Data were analyzed using Statistical Product and Service Solutions 26.0 (releases 2019, IBM Corp., Armonk). Count data are presented as frequency and percentage. The chi-square test was used to analyze the changing trend of the positive rate. Pairwise comparisons were made and the Bonferroni method was employed to correct the test level. A *P* value <.05 was considered statistically significant.

## 3. Result

### 3.1. Comparison of overall positivity rates for respiratory infections (2018–2024)

**Figure 1.**
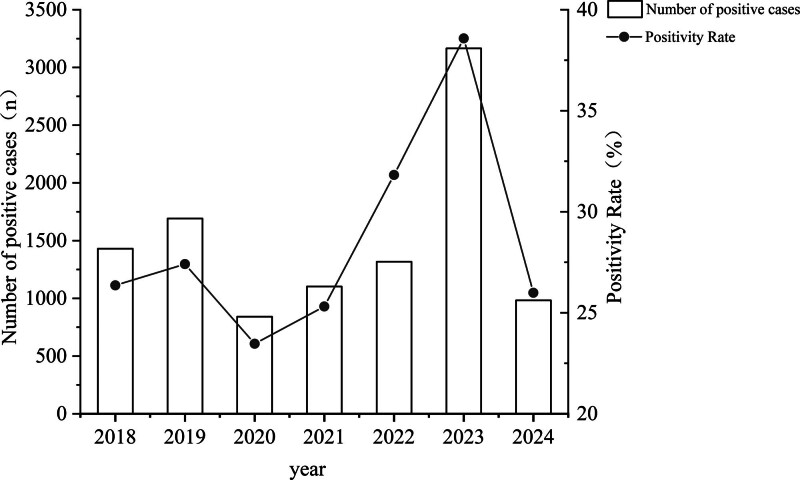
The annual changing trend of the positive rate among patients with respiratory tract infections from 2018 to 2024. The bar chart represents the number of positive respiratory pathogen antibodies in the screen.

From 2018 to 2024, we enrolled 35,665 patients, of whom 10,531 were positive for antibodies against at least 1 pathogen, with an overall positivity rate of 29.53% (10,531/35,665). The annual positivity rates significantly fluctuated: 26.36% (1430/5425) in 2018, 27.41% (1692/6173) in 2019, 23.47% (841/3584) in 2020, 25.31% (1103/4358) in 2021, 31.82% (1317/4139) in 2022, and 38.58% (3165/8204) in 2023, followed by 25.99% (983/3782) in 2024. The positivity rate (23.47%) in 2020 was significantly lower than that in 2018 (26.36%) and 2019 (27.41%). The positivity rates observed in 2022 (31.82%) and 2023 (38.58%) were more than those observed from 2018 to 2021 (range: 23.47–27.41%). These differences reached statistical significance (χ^2^ = 496.188, *P* < .001), with notable variations between the positivity rate (25.99%) in 2024 and the preceding years (Fig. 1).

### 3.2. Comparative analysis of pathogen positivity rates (2018–2024)

**Figure 2.**
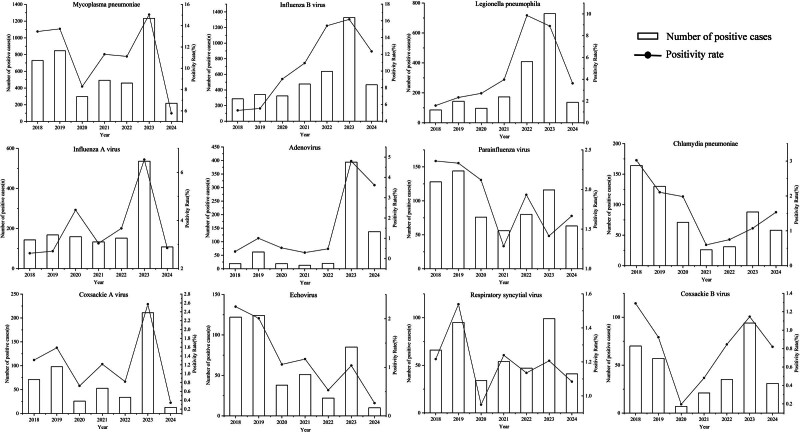
Comparative analysis of the positivity rate of pathogens causing respiratory tract infections from 2018 to 2024. The bar chart represents the detection quantity of various pathogens (left axis), and the line chart represents the positive rate (right axis).

From 2018 to 2024, mycoplasma pneumoniae exhibited the highest overall detection rate (11.99%, 4278/35,665), followed by influenza B virus (10.83%, 3861/35,665), influenza A virus, respiratory syncytial virus, parainfluenza virus, chlamydia pneumoniae, legionella pneumophila, coxsackie A virus, coxsackie B virus, echovirus, and adenovirus had relatively low positivity rates, ranging from 0.88% to 4.97%. Comparative analysis of annual pathogen-specific rates revealed distinct epidemiological patterns: For mycoplasma pneumoniae, the positivity rate in 2020 (8.29%) was significantly lower than that in 2019 (13.70%), while the positivity rates in 2021 (11.31%) and 2022 (11.11%) were significantly more than that in 2020. The rate peaked in 2023 (15.04%) and decreased in 2024 compared to 2018 to 2023 (χ^2^ = 290.945, *P* < .001). The positivity rates of influenza B virus showed progressive increases from 2018 to 2023 and decreased in 2024 (χ^2^ = 706.350, *P* < .001). The positivity rate of legionella pneumophila showed an initial rise in 2021 compared to 2018 to 2020. It continued to increase in 2022 to 2023 but decreased in 2024 compared to 2022 to 2023 (χ^2^ = 760.260, *P* < .001). Influenza A virus displayed the highest positivity rate in 2023 (6.53%), significantly exceeding that in all other years (χ^2^ = 219.228, *P* < .001). Similarly, adenovirus peaked in 2023 (4.80%) and 2024 (3.62%), with rates substantially higher than in preceding years (χ^2^ = 681.293, *P* < .001). The complete pathogen-specific case numbers and detection rates are presented in Figure 2.

### 3.3. Age distribution patterns of respiratory infections (2018–2024)

**Figure 3.**
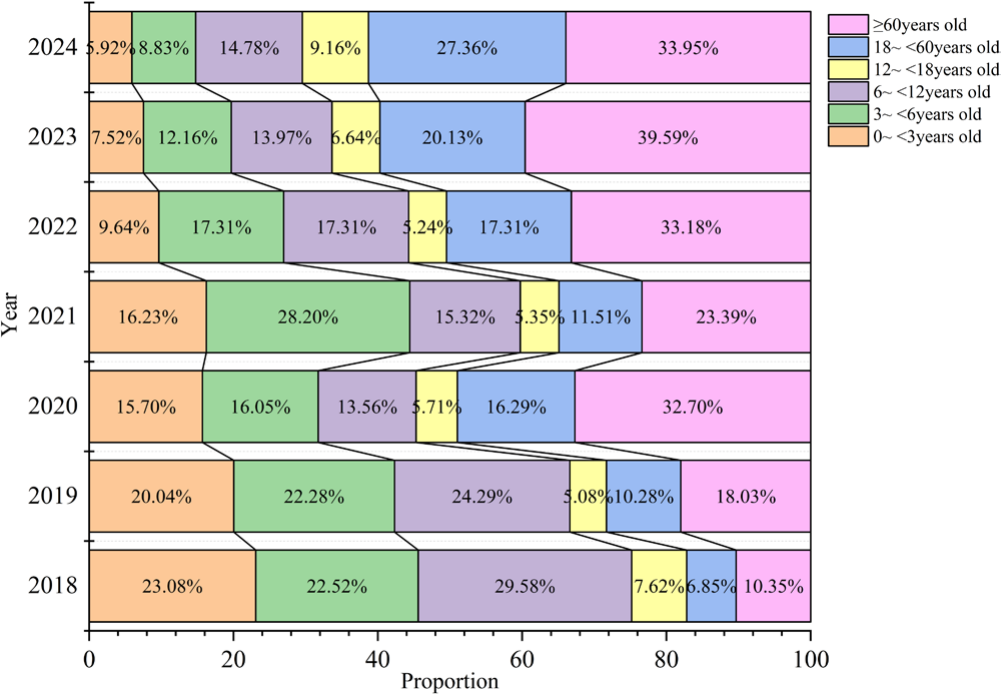
Changes in the age distribution of patients with respiratory tract infections from 2018 to 2024. The horizontal coordinate represents the proportion of different age groups among positive cases. Including 0 to <3 yr (infancy/toddler group), 3 to <6 yr (preschool group), 6 to <12 yr (school-age group) 12 to <18 yr (adolescent group), 18 to <60 yr (adult group), ≥60 yr (elderly group), the vertical coordinate represents each year from 2018 to 2024.

The 10,531 positive cases were distributed in the following groups: 13.32% (1403/10,531) in the 0 to <3 years group, 17.52% (1845/10,531) in the 3 to <6 years group, 18.35% (1932/10,531) in the 6 to <12 years group, 6.37% (671/10,531) in the 12 to <18 years group, 15.86% (1670/10,531) in the 18 to <60 years group, and 28.58% (3010/10,531) in the ≥60 years group. The 0 to <3 years group showed significantly lower proportions in 2020 and 2021 compared to 2018 (χ^2^ = 350.647, *P* < .001), with further reductions in 2022 to 2023 compared to 2018 to 2021, and the lowest rates observed in 2024 versus 2022. The 3 to <6 years group exhibited decreased prevalence in 2020 compared to 2018 and 2019 (χ^2^ = 253.503, *P* < .001), peaked in 2021 compared to 2018 to 2020, and then progressively decreased in 2022 to 2024. The 6 to <12 years group displayed reduced proportions in 2019 compared to 2018 (χ^2^ = 229.986, *P* < .001), followed by a sustained decrease in 2020 to 2024 compared to 2019. The 18 to <60 years group showed an initial increase in 2018 to 2020, a temporary decline in 2021 (χ^2^ = 284.874, *P* < .001), subsequent increases in 2022 to 2023, and a peak prevalence in 2024. The ≥60 year group exhibited increasing prevalence in 2018 to 2020 (χ^2^ = 562.307, *P* < .001), and a transient decrease in 2021 compared to 2020. In this group, the rates elevated in 2022 to 2023 compared to 2021, and finally decreased in 2024 compared to 2023 (Fig. 3).

### 3.4. Changes in respiratory infection patterns (2018–2024)

**Figure 4.**
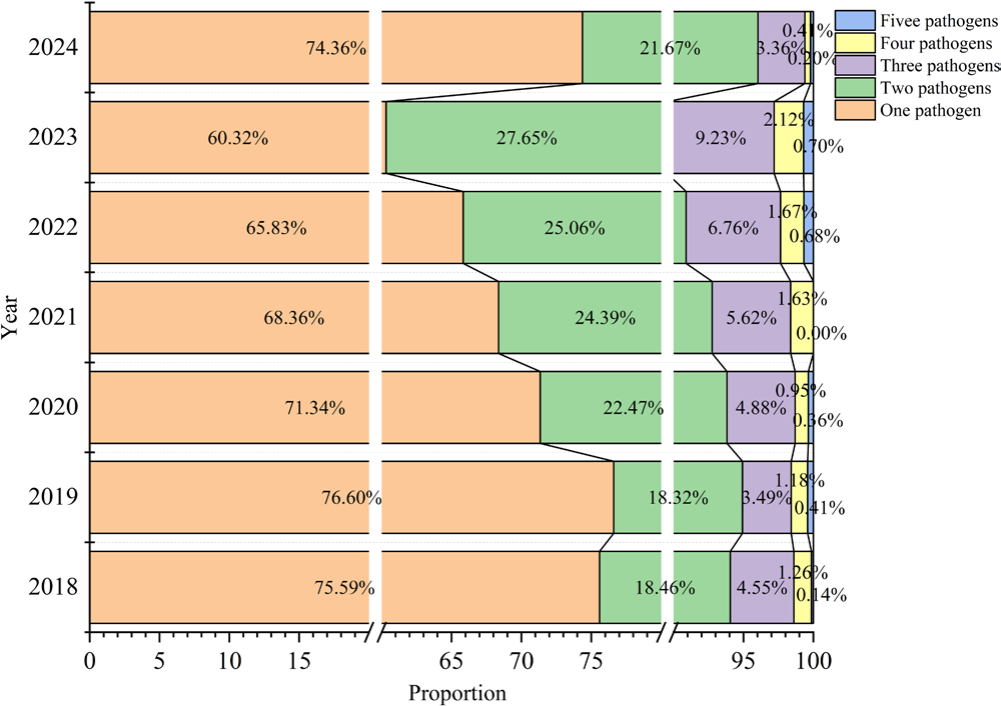
Changes in respiratory infection patterns from 2018 to 2024. The horizontal axis represents the proportion of different infection patterns in positive cases, including 5 groups: 1 pathogen infection, 2 pathogen infection, 3 pathogen infection, 4 pathogen infection, and 5 pathogen infection. The vertical axis represents each year from 2018 to 2024.

Among the 10,531 positive cases, single-pathogen infections predominated (68.73%, 7238/10,531), followed by dual-pathogen coinfections (23.26%, 2450/10,531). The prevalence rates for triple-, quadruple-, and quintuple-pathogen coinfections were 6.09% (641/10,531), 1.49% (157/10,531), and 0.43% (45/10,531), respectively. Single-pathogen infection rates significantly decreased in 2022 compared to 2018 to 2021 (χ^2^ = 206.732, *P* < .001) and declined further in 2023 compared to 2022. Single-pathogen infection rate increased in 2024 and exceeded that in both 2022 and 2023. Dual-pathogen coinfection rates increased in 2021 to 2023 compared to 2018 and 2019 (χ^2^ = 80.521, *P* < .001); however, it decreased in 2024 compared to 2023. For triple-pathogen coinfections, the prevalence increased in 2022 compared to 2019 (χ^2^ = 96.939, *P* < .001). It further increased in 2023 compared to 2018 to 2021, but then decreased in 2024 compared to 2022 to 2023. Quadruple-pathogen co-infection rates exhibited a significant decrease in 2024 compared to 2023 (χ^2^ = 20.040, *P* = .003). Quintuple-pathogen coinfections exhibited consistently low prevalence without significant variations between years (*P* > .05; Fig. 4).

## 4. Discussion

In this study, the higher positivity rates of respiratory infections in 2022 and 2023 compared to 2018 to 2021 may be attributed to the “immunity debt” phenomenon. Prolonged pandemic control measures limited population exposure to various respiratory pathogens, resulting in diminished preexisting immunity. After lifting the restrictions, this immune-naive status increased susceptibility to multiple pathogens.^[4]^ The subsequent normalization of positivity rates by 2024 further supports this immunity debt hypothesis. Notably, the significantly higher positivity rates in 2022 compared to those in 2020 and 2021 likely reflect both the stringent early-pandemic measures (mask-wearing, social distancing) that effectively prevented the transmission of conventional respiratory pathogens.^[5–7]^ It may also be due to potential underdiagnosis due to healthcare avoidance among mild cases during the pandemic.

Pathogen-specific analysis revealed markedly decreased detection rates in 2020 to 2022 for mycoplasma pneumoniae, parainfluenza virus, chlamydia pneumoniae, echovirus, adenovirus, and coxsackievirus A/B, demonstrating the broad impact of Coronavirus Disease 2019 containment measures on the epidemiology of respiratory pathogens. Conversely, post-pandemic surges were observed for mycoplasma pneumoniae, influenza A/B viruses, and adenovirus. Notably, the mycoplasma pneumoniae outbreak in 2023 was not caused by novel strains or emergent macrolide-resistant variants,^[8]^ suggesting delayed transmission of preexisting strains due to pandemic controls rather than viral evolution. This finding further highlights the importance of preventive measures.

Of particular concern is the persistent upward trend in the detection rates of legionella, consistent with Centers for Disease Control and Prevention surveillance, which showed a 9-fold increase in reported United States of America cases (2000–2018).^[9]^ Underdiagnosis may suggest that the actual incidence is 1.8 to 2.7 times higher.^[10]^ Unlike droplet-transmitted pathogens, the environmental transmission of legionella was not affected by pandemic measures.^[11]^ Building closures may have exacerbated risk through stagnant water systems, inadequate disinfection, and optimal biofilm conditions.^[12]^ However, the indirect immunofluorescence method was used to detect antibodies against Legionella in this study, which has a certain false positive rate. According to the data provided by the manufacturer, the false positive rate is approximately 16.5%, which may falsely increase the number of those with Legionella infections. However, its annual increasing trend should be given sufficient attention.

Age-stratified analysis showed post-pandemic increases in elderly (≥60 years) positive cases and decreased pediatric cases (0 to <12 years). This suggests greater vulnerability of the elderly population after the Coronavirus Disease 2019 pandemic, potentially reflecting age-related immune dysregulation, including impaired viral clearance, hyperinflammation, and antigen presentation abnormalities.^[13]^

From the perspective of infection patterns, our data demonstrated a progressive increase in multiplex pathogen positivity rates between 2018 and 2023. These findings corroborate the observations of Yi Li et al from Peking Union Medical College Hospital regarding increased respiratory pathogen co-infection rates in 2023.^[14]^ This epidemiological pattern likely originates from the synergistic interaction of 2 principal mechanisms. First, respiratory coinfections are intrinsically associated with host immune competence and pathogen coexistence dynamics.^[15]^ Sustained pandemic containment measures over 3 consecutive years may have contributed to population-wide immune attenuation and antibody level reduction. Furthermore, Severe Acute Respiratory Syndrome Coronavirus 2 induced immune perturbation can potentially increase host vulnerability to the invasion of secondary pathogens. Second, the temporal convergence of multiple pathogen transmission seasons establishes an epidemiological nexus favorable for coinfections.^[16]^ The approximate synchrony of mycoplasma pneumoniae, influenza virus, and legionella epidemic periods observed in our study^[17–19]^ is consistent with significant post-epidemic surges in pathogen-specific IgM seropositivity. This phenomenon may be attributable to persistent IgM from previous infections coinciding with subsequent exposures to different pathogens, thereby increasing multiplex detection rates.

In conclusion, our findings showed dynamic shifts in respiratory pathogen epidemiology affected by public health interventions. Targeted measures for high-risk groups (elderly and children), guided by ongoing surveillance, are crucial to reduce the disease burden on families and healthcare systems.

## 5. Limitations

This study has certain limitations. Firstly, we only included clinical samples from a single center, limiting the generalizability of our findings. In addition, the results of the antibody test might be affected by factors such as the disease course at the time of sample collection, vaccination, and the presence of severe immunodeficiency. Therefore, there might be a certain degree of false positive results.

## Acknowledgments

The authors would like to express their gratitude to EditSprings (https://www.editsprings.cn) for the expert linguistic services provided.

## Author contributions

**Conceptualization:** Lei Zhang.

**Data curation:** Junshuai Ma.

**Investigation:** Shaobo Qu.

**Methodology:** Mingfu Cao.

**Project administration:** Zhangxin Cui.

**Writing – original draft:** Lei Zhang.

**Writing – review & editing:** Mingfu Cao.
